# Correction

**DOI:** 10.1080/26410397.2026.2751901

**Published:** 2026-11-04

**Article title:** Internet use, exposure to digital family planning messages, and sexual agency among partnered women in Northern Nigeria: implications for digital family planning intervention

**Authors:** David Aduragbemi Okunlola, Oluwatobi Abel Alawode, Abayomi Folorunso Awoleye, Benjamin Bukky Ilesanmi

**Journal:** *Sexual and Reproductive Health Matters*

**Bibliometrics:** Volume 31, Number 4, pages 1-27

**DOI:** http://dx.doi.org/10.1080/26410397.2023.2261681

When this article was first published, changes to references during the proofing stage resulted in incorrect citation numbers in the text. This has now been corrected.

